# CircRNA-Associated ceRNA Network Reveals Focal Adhesion and Metabolism Pathways in Neuropathic Pain

**DOI:** 10.1155/2022/7246904

**Published:** 2022-08-27

**Authors:** Kun Wang, Jun-Ping Bao, Zhi-Min Zhou, Lu Mao, Xiao-Tao Wu

**Affiliations:** ^1^Department of Orthopedics, Zhongda Hospital, Southeast University, Nanjing, China; ^2^Medical School of Southeast University, Nanjing, China; ^3^School of Biomedical Engineering and Informatics, Nanjing Medical University, Nanjing, China

## Abstract

**Background:**

Increasing evidence has shown that noncoding RNAs perform a remarkable function in neuropathic pain (NP); nonetheless, the mechanisms underlying the modulation of competitive endogenous RNA in NP remain uncertain. The goal of this research was to investigate the molecular processes underlying NP.

**Methods:**

We utilized the Gene Expression Omnibus (GEO) to obtain NP-related microarray datasets that included the expression patterns of circular RNAs (circRNAs) and messenger RNAs (mRNAs). Following that, bioinformatics analyses and a molecular biology experiment were carried out.

**Results:**

According to the findings, carrying out enrichment studies of the targeted genes had an impact on a variety of NP-related pathways. Notably, we isolated a ceRNA subnetwork incorporating two upregulated circRNAs (Esrrg and Map3k3) which primarily participate in the focal adhesion pathway by regulating Integrin Subunit Beta 4 (ITGB4) and two downregulated circRNAs (Dgkb and Atp2a2), which potentially regulate metabolism-related molecule Lipase A (LIPA).

**Conclusions:**

According to our findings, the focal adhesion and metabolic signaling pathways could be critical in the advancement of NP, and some circRNA might regulate this biological process through the ceRNA network, which might offer pertinent insights into the underlying mechanisms.

## 1. Background

Neuropathic pain (NP) is the most frequently reported symptom of lumbar disc herniation. The most important pathogenic mechanism is the formation of multiple inflammatory responses mediated by the spinal nerve roots, which are triggered by the tensile stress on the bulging disc [1]. To treat this condition, surgical procedures, including lumbar interbody fusion and discectomy, are now being employed, in addition to nonsurgical options such as medications and active physiotherapy [2]. Nonetheless, their overall effectiveness is low due to the fact that the etiology of NP is still not fully understood. It is thus imperative that the exact mechanisms of NP progression be investigated and that early intervention approaches and medication treatment targets be identified as soon as possible.

Circular RNAs (circRNAs) are noncoding RNAs characterized by closed continuous looped structures and are found in abundance across mammalian tissues. CircRNAs have higher stability as opposed to linear RNAs because they do not have terminal 5′ and 3′ ends [3]. CircRNAs are important players in the pathogenic progression of NP. Cao and his colleagues were the first to discover circRNAs in a sciatic nerve model of chronic constriction injury (CCI). The researchers identified 469 circRNAs that were differentially expressed between the mice that had CCI and those that received sham operations [4]. By using the sequencing technology, Zhou et al. were able to identify the noncoding RNA expression profile associated with NP following retention nerve damage. The findings illustrated that the microRNAs (miRNAs) and 188 circRNAs expression levels had been dramatically altered [5]. A recent study conducted by Wang and his colleagues discovered that miRNA-124 binds to circHIPK3 and modulates the proinflammatory mediators, such as interleukin-1 beta (IL-1*β*), interleukin-6 (IL-6), interleukin-12 (IL-12), and tumor necrosis factor-alpha (TNF-*α*), and that intrathecal administration of circHIPK3 shRNA can be applied in the treatment of NP in rats with diabetes [6]. The role of circRNAs in NP has been underlined by these investigations; nevertheless, the expression and function of the vast majority of circRNAs in this disease condition remain unclear at this time. Furthermore, circRNAs are illustrated to be abnormally expressed in numerous pathogenic illnesses. We can better understand the pathological mechanisms of NP by building competitive endogenous RNA (ceRNA) networks.

**Figure 1:**
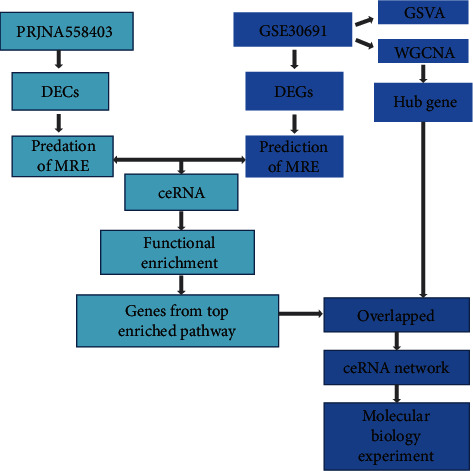
Flowchart of circRNA-related ceRNA regulatory network analysis in NP.

In this study, some novel circRNAs may act as ceRNA to regulate gene expression in NP, and their potential mechanisms have been investigated by utilizing circRNA and gene expression dataset collected from GEO database, and several bioinformatic tools were used. The findings of the bioinformatic analysis were validated by a molecular biology experiment. The flowchart in this study is shown in Figure 1. First, datasets of NP were obtained from the GEO database, and differentially expressed circRNAs (DECs) and genes were also acquired. Then, to demonstrate whether the DECs function as ceRNAs in NP, their related miRNAs and miRNA target genes have been collected, and a circRNA/miRNA/mRNA network also has been constructed. Furthermore, to validate whether the target genes or pathways enriched with target genes in the ceRNA network were significantly altered in gene expression level, GSVA and WGCNA analyses were performed. Results from both methods were consistent with the finding that ECM interaction or focal adhesion-related pathways were with elevated activity after spinal nerve ligation, while metabolism-related especially fatty acid metabolism was downregulated. These pathways were also found to be circRNA target through ceRNA network. Then the target genes from the top enriched pathway like focal adhesion and metabolism pathway were used to construct a potential circRNA-related ceRNA network and the hub genes detected by WGCNA were highlighted in the network. We validate the potential regulation between 4 circRNAs (Esrrg, Map3k3, Dgkb, and Atp2a2) and 2 hub genes which were also circRNA-related ceRNA target genes like ITGB4 and LIPA by molecular biology experiment. Based on this analysis flow, our study broadens the understanding of the mechanisms of NP occurrence and provides avenues for developing potential therapeutic interventions for NP.

## 2. Materials and Methods

### 2.1. GEO Datasets

For circRNA, the raw data were obtained from the NCBI SRA with the accession number PRJNA558403. STAR was used to translate reads to the reference genome *Rattus norvegicus* (UCSC rn6), which was used to detect and quantify circRNAs. The circRNA was quantified and annotated with the aid of the CIRCexplprer2 program. As to gene expression, data for GSE30691 were downloaded and normalized using the “RMA” method, and probes with low expression levels were removed from the analysis. After that, only specimens from the spinal nerve ligation cohort were utilized in the subsequent analyses. The flowchart of the analysis is shown in Figure 1.

### 2.2. Analysis of Differential Expressions

NOIseq was utilized to analyze the differentially expressed circRNAs (DECs) employing settings as follows: replicates = “no” and lc = 1 [7]. DECs were those with a threshold of *q* = 0.8. Because circRNAs with elevated expression are regarded as significant, circRNAs having an absolute ranking value in the topmost ten highest expressions and a *q* value ≥ 0.6 were also determined to be significantly DECs. The “limma” function in *R* was utilized to conduct the analysis of differentially expressed mRNAs. In order to be termed differentially expressed, probes had to attain a *P* value < 0.05 and an absolute fold-change ≥ 1.5.

### 2.3. Functional Annotation

The Kyoto Encyclopedia of Genes and Genomes (KEGG) database and Gene Ontology (GO) category database were used for functional annotation of genes. Only those GO categories or pathways containing at least 5 DEGs were kept for further analysis. Enrichment analysis of GO categories was performed by *R* cluster Profiler (v3.14.3) package, and enrichment analysis of pathways was tested upon hypergeometric distribution by *R* “phyper” function. Those GO categories with a false discovery rate (FDR) < 0.05 were considered as significantly enriched. While pathways with a *P*-value < 0.05 were regarded as enriched.

### 2.4. CircRNA-miRNA-mRNA Network

MiRanda was used to predict the possible miRNA binding domains to all of the DECs using the default settings. Targetscan (https://www.targetscan.org/vert_71/) and miRanda were utilized to acquire the microRNA targets. Subsequently, only the targets that were differentially expressed were selected. Premised on the ceRNA hypothesis, we chose circRNA as ceRNA. CeRNA interactions were defined as any circRNA and mRNA pair that had a similar regulatory direction (both up/downmodulation across two groups of specimens) and that was bound by a similar miRNA. Utilizing the ceRNA network, Cytoscape 3.6.1 program was used to retain the target genes of chosen pathway-related genes and play them out in a simulation.

### 2.5. GSVA for Gene Expression Data

Gene set variation analysis (GSVA) is a method that estimates the variation of pathway activity over a sample population in an unsupervised manner [8]. This analysis was performed by *R* ′ GSVA′ package. Pathways collected from the KEGG database were used. The statistical difference of pathway activity across time series was calculated by *R* “limma” package. Pathways with a *P*-value<0.05 were considered as differential activated across time series.

### 2.6. WGCNA for Gene Expression Data

Weighted correlation network analysis (WGCNA) was performed by *R* package WGCNA [9]. All the expressed genes other than DEGs were used. According to the principle of scale-free networks, the weighting coefficient *β* was determined as 16 by using the integrated function (pickSoftThreshold) in the WGCNA package. The network type set as “signed” and “bicor” (bi-weighted correlation) was used to calculate the correlation adjacency matrix. Co-expressed gene modules were identified by using a dynamic tree cut with the following major parameters: minModule Size of 20 and deepSplit of 2.

### 2.7. Dorsal Root Ganglion (DRG) Culture and Treatment

Southeast University Laboratory Animal Center provided the bilateral lumbar L1-L6 DRGs, which were derived from fifty male adult Sprague–Dawley rats that weighed between 200 and 220 g. After dissecting the DRGs, they were digested enzymatically for 50 minutes at 37°C with 0.25% trypsin. We then conducted mechanical dissociation and plating operations. After that, we placed the neurons in 6-well plates and cultured them in a culture medium (ThermoFisher, Shanghai, China) that contained 2 mM l-glutamine, B27, Neurobasal®-A media, as well as the antibiotics streptomycin and penicillin. Throughout the tests, the cells were preserved at 37°C in an incubator with 5% CO_2_. All succeeding experiments were followed by a one-week cell culture period. In the experiment, 1 *μ*g/ml LPS was used to simulate the in vitro inflammatory pain model. All the experimental procedures were evaluated and subjected to approval from the Institutional Animal Care and Use Committee of Southeast University School of Medicine (NO.20190218002) following the guidelines provided by the committee.

### 2.8. Plasmid Construction and Small Interfering RNA (siRNA) Transfection

In vitro DNA synthesis was conducted to clone the full-length hsa circ Dgkb and hsa circ Atp2a2 into the pLCD5H-ciR plasmid, resulting in the construction of the hsa circ Dgkb and hsa circ Atp2a2 overexpression vectors. For the negative control (NC) in this experiment, we utilized the empty pLCD5H-ciR plasmid. To achieve optimal results, transfection of the plasmids was initiated when DRG cells attained a confluence of 30 to 50 percent utilizing Lipofectamine™ 3000 Transfection Reagent (Invitrogen, Shanghai, China). The siRNA targeting circEsrrg and circMap3k3 were obtained from GeneChem (China). Sequences of the CircEsrrg (siRNA) included: sense strand 5′-GGTGTCTGCTCCCTGTATTAT-3′, and sequences of the CircMap3k3 (siRNA) included: sense strand 5′-ACCTCTCTGTCAGAGAACGGT-3′. The siRNAs were transiently transfected into cells using LipofectamineVR RNAiMAX Transfection Reagent (Invitrogen) following the guidelines stipulated by the manufacturer.

### 2.9. Quantitative Real-Time Polymerase Chain Reaction (qRT-PCR) Analysis

In accordance with the recommendations provided by the manufacturer, Trizol (Thermo Fisher Scientific, Inc.) was utilized to isolate total RNA from LPS-treated and Sham group DRG specimens, followed by reverse transcription of 1 *μ*g of total RNA into first‐strand cDNA utilizing a PrimeScript RT Reagent kit (Takara Bio, Inc., Otsu, Japan). Subsequently, the SYBR‐Green real‐time PCR kit (Thermo Fisher Scientific, Inc.) was utilized to conduct qPCR with the help of the ABI StepOnePlus Real-Time PCR system (Applied Biosystems; Thermo Fisher Scientific, Inc.). The internal control for the analysis of circulating RNAs was chosen as GAPDH. In order to prepare the reactions, the following conditions were utilized: 2 *μ*l cDNA, 5 *μ*l RNase-free water, 0.125 *μ*l reverse primer, 0.125 *μ*l forward primer, 0.25 *μ*l ROX Reference dye II, and 7.5 *μ*l SYBR Premixm Ex Taq II. The following were the thermocycling parameters: 30 seconds for one step at 95°C, accompanied by 40 cycles of 95°C for 5 seconds and 60°C for 30 seconds, and the last step at 95°C for 15 seconds, 60°C for 15 seconds, and followed by 95°C for 15 seconds. In this analysis, the 2^−ΔΔcq^ method was adopted to determine the circRNAs' relative expression level [10].  Table 1 provides a list of the primer sequences.

### 2.10. Western Blot

Isolating the total proteins from DRG was accomplished utilizing RIPA lysis buffer. To measure the protein concentrations of the samples, the BCA Protein Assay kit was utilized. After separating all of the proteins utilizing sodium dodecyl sulfate-polyacrylamide gel electrophoresis (SDS-PAGE), they were loaded onto the polyvinylidene fluoride (PVDF) membranes. After being blocked for hours in 5 percent nonfat milk, the membranes were subjected to overnight incubation at 4 degrees Celsius with the primary antibodies. Incubation with secondary antibody for 1 hour was performed after the membranes had been rinsed using Tris-buffered saline with Tween 20 (TBST). The following primary antibodies were used: anti-ITGB4 (Bioss, 1 : 500, cat: bs-10028R); anti-LIPA (Proteintech, 1 : 1000, cat:12956-1-AP). Secondary antibody was used: goat anti-rabbit (Servicebio, 1 : 2000, cat: G1213-100*μ*l). By using a Chemi-doc Gel Imaging System (Bio-Rad), we visualized Western blots by enhanced chemiluminescence. Analyses were conducted with Image Lab (RRID : SCR_014210) and ImageJ (National Institutes of Health, RRID : SCR_003070) software. *β*-Actin served as an internal reference.

### 2.11. Statistical Analysis

One-way ANOVA with a post hoc Tukey method was used to determine the significance of differences in multiple comparisons. An unpaired Student's *t*-test was used to compare two unmatched groups. Pearson's correlation coefficients were calculated to investigate the association between indicated parameters. *P* values < 0.05 were considered statistically significant.

## 3. Results

### 3.1. Expression Profiles of circRNAs and Analyses of the Functional Enrichment of the Parental Gene

**Figure 2:**
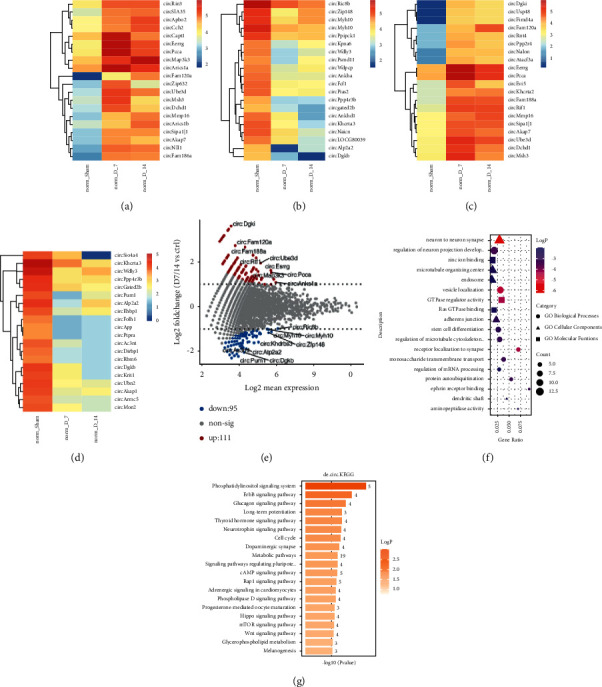
Screening of DECs and enrichment analysis of related gene function. (a)–(d) Heat maps of significant DECs. Each circRNA is represented by the corresponding gene name. Red represents higher expression, and blue represents lower expression. (e) Scatter plot of differentially expressed genes, with a total of 111 upregulated and 95 downregulated genes. Each dot represents one circRNA, with red dots representing upregulation, blue dots representing downregulation, and grey dots representing nondifferentially expressed molecules. Significant DECs are simultaneously labeled with names. (f) GO enrichment analysis. The *P* value is shown from red to blue for the smallest to largest. The circle size is shown from large to small based on the number of different related genes. (g) KEGG pathway enrichment analysis. The horizontal axis represents −*log*10 (*P* value), and the colors are gradient-filled to indicate the −*log*10 (*P* value). The number on the right represents the number of differences.

We retrieved NP-related circRNA expression datasets by RNA-seq analysis from SRA database PRJNA558403 and performed data preprocessing (S1). After that, we conducted bioinformatics investigations. Each of the circRNA is linked to the name of its matching parental gene in the heatmaps. To find DECs that were statistically significant, two approaches were applied. The topmost 20 upmodulated and downmodulated molecules based on fold change are separately shown (Figures 2(a) and 2(c)). The topmost 20 upmodulated and downmodulated results based on statistical analysis are independently illustrated (Figures 2(b) and 2(d)). On the scatter plot, there are 111 DECs that have been upmodulated and 95 DECs that have been downmodulated (Figure 2(e)). Functional enrichment analysis of the parental genes of these DECs showed these DEC-associated genes are mostly involved in signal transduction, neuronal projections, and adhesion (Figures 2(f) and 2(g)). As a consequence of these findings, DECs might be engaged in these biological activities.

### 3.2. Profiling and Enrichment of mRNA Expression

**Figure 3:**
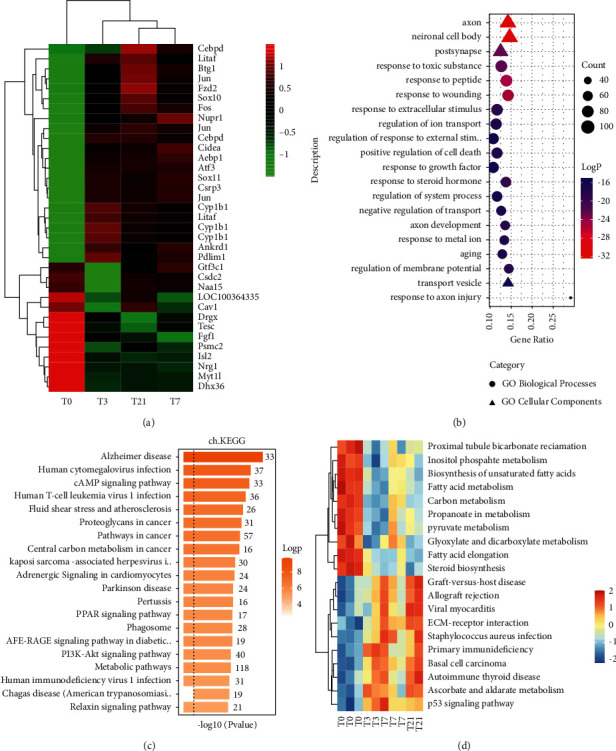
mRNA expression profiling and enrichment analysis. (a) Heatmap of differentially expressed transcription factors (fold change>2.0). (b) GO enrichment analysis. The *P* value is shown from small to large in red to blue. The circle size is shown from largest to smallest based on the number of differentially expressed genes. BP and MF are represented by three different shapes. (c) KEGG pathway enrichment analysis. Relatively different genes are enriched in the top-ranked pathways. (d) Heatmap of the scaled GSVA score of KEGG pathways with variation during the time course. The color blue to red represents a gradient from low to the high score.

Due to the possibility that circRNAs are implicated in the transcription modulation and stability of disease-related genes, we obtained NP-associated gene expression microarray datasets from GSE30691 and performed data preprocessing on the data (S2). After that, we undertook a bioinformatics analysis. The transcriptional factors that were found to be differentially expressed with fold change values greater than1 over time are represented in the heatmap (Figure 3(a)). Functional enrichment analysis of GO and KEGG pathways was performed, and it was discovered that some of the mRNAs with differential expression were implicated in biological processes, including axonal, and signaling, as well as metabolic processes (Figures 3(b) and 3(c)). The above found the pathways related to NP process from the perspective of differential genes. Furthermore, we investigate the altered pathway activity profile from the perspective of gene set enrichment level by GSVA analysis. Pathways with *P*-value < 0.05 were considered as statistically altered during NP and plotted as a Heatmap. Based on the findings, metabolism-associated signaling pathways exhibited a decreasing pattern over time, but the inflammatory reaction and ECM-associated signaling pathways exhibited an upward pattern over time (Figure 3(d)).

**Figure 4:**
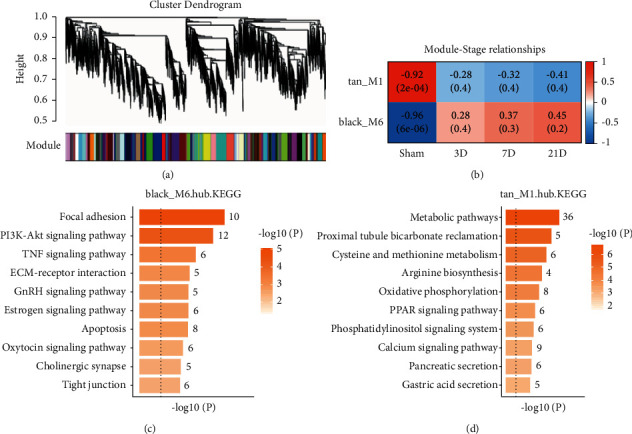
WGCNA analysis of gene expression. (a) Gene dendrograms of coexpressed gene modules. Genes belonging to the same module were in the same color. (b) Heat map of the correlations between module eigengene (the first principal component of the expression matrix of the corresponding module) and time traits, with positive correlation in red and negative in blue. When the positive correlation is high, it means the eigengene increases with increasing time. (c)-(d) KEGG pathway enrichment of hub genes belongs to the black and tan module.

Then weighted correlation network analysis (WGCNA) was used to detect coexpressed gene module during NP and hub genes were identified. In total, 36 modules were identified. Genes belonging to the same module were colored in the same color (Figure 4(a)). Genes in the black and tan module showed significantly differential expressed during NP (Figure 4(b)). This indicates that genes in these two modules may be responsible for NP process. Hub genes are those that have high connectivity in the coexpressed network, which play a vital role in biological processes and influence the regulation of other genes in related pathways [11]. We defined the gene with a threshold value of module membership (kME) greater than 0.8 and differential expressed during NP as hub genes [9]. Finally, 305 genes from the black and tan modules were regarded as the hub gene. By functional enrichment analysis, these hub genes in the black module which has an elevated expression profile were enriched with a focal adhesion pathway (Figure 4(c)), while hub genes in the tan module which has a downregulated expression profile were enriched with metabolism pathway (Figure 4(d)).

### 3.3. Analysis of mRNAs and circRNAs Using ceRNA

**Figure 5:**
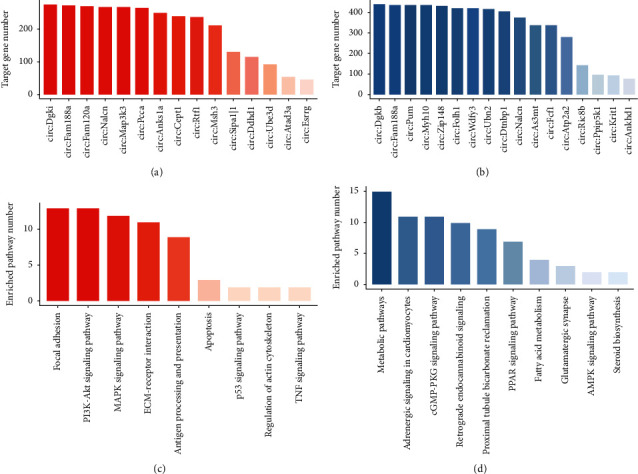
CircRNA target analysis upon ceRNA pattern. (a) Number of target genes corresponding to upregulated circRNAs and colored in red. The larger in size of number the deeper in color. *Y*-axis represents the number of target genes. (b) Number of target genes corresponding to downregulated circRNAs and colored in blue. (c) Frequency of top5 enriched KEGG pathways of upregulated circRNAs' target genes and colored in red; the larger in size of the frequency, the deeper in color. *Y*-axis represents the frequency of top5 enriched pathways. (d) Frequency of top5 enriched KEGG pathways of downregulated circRNAs' target genes and colored in blue.

After performing a fundamental study of circRNAs in the preceding section, we anticipated numerous circRNAs that may participate in disease-associated biological processes through constructing ceRNA regulatory network. Any differentially expressed circRNAs and mRNAs (abbreviated as DEcircRNAs and DEmRNAs) pair that had a similar regulatory direction (both up/downmodulation across two groups of specimens) and that was bound by a similar miRNA was defined as a ceRNA interactions. Although the lack of miRNA expression level, this analysis provided the potential ceRNA regulatory network between circRNA and mRNA. Multiple target genes were shown to correspond to the upmodulated and downmodulated DECs, as demonstrated by the histograms. When the number of targets of DECs increases, it indicates that the DECs are becoming more relevant to regulate NP-related biological processes (Figures 5(a) and 5(b)). Following that, we conducted pathway enrichment analysis on target genes that corresponded to the DECs, each DECs were calculated separately. The top 5 enriched pathways of these DECs were collected and the frequency of these top enriched pathways was shown as a histogram (Figures 4(c) and 4(d)). Focal adhesion was the most enriched pathway for these upregulated DECs and the metabolism pathway for downregulated DECs. From the WGCNA analysis, we learned focal adhesion and metabolism pathway were enriched with hub genes during NP. From this part of analysis, we inferred these DECs may regulate NP by targeting focal adhesion and metabolism pathway through a ceRNA-mediated mechanism.

### 3.4. Establishment of the ceRNA Network and Confirmation of Chosen circRNAs through qRT-PCR

**Figure 6:**
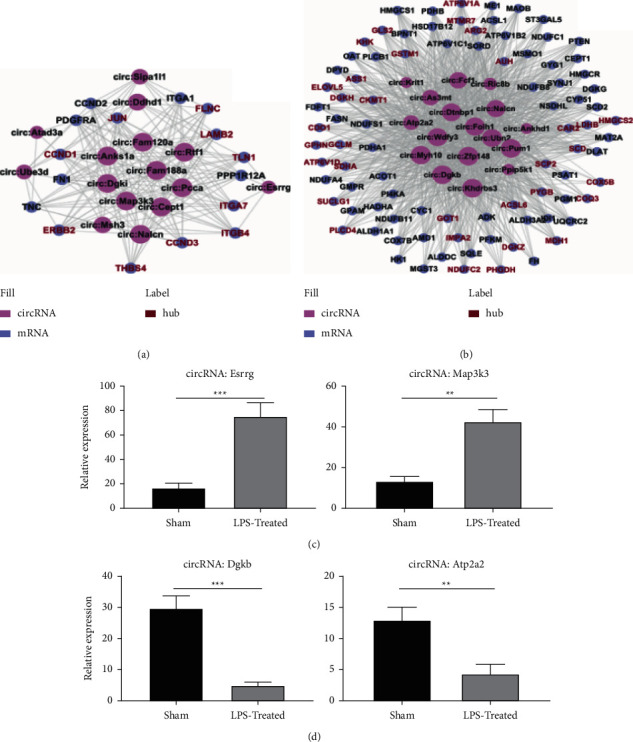
CircRNA-mRNA network and validation results for the selected circRNAs. (a) Upregulated circRNA and focal adhesion pathway-related mRNA networks. CircRNAs are filled in red and mRNAs in purple, hub gene from WGCNA analysis are labeled in dark red color, the size of the node is based on the number of outdegree, with a larger size indicating a larger in outdegree. (b) Downregulated circRNA and metabolism pathway-related mRNA networks. (c)-(d) Quantitative reverse transcription-polymerase chain reaction analysis. Two upregulated and 2 downregulated circRNAs demonstrated to be consistent with the ceRNA network results (^*∗∗*^*P* < 0.01, ^*∗∗∗*^*P* < 0.001 compared to the sham group, *n* = 6/group, an unpaired Student's *t*-test).

We evaluated the genes of the top enriched pathway to build the potential ceRNA network, where the DEGs for focal adhesion were chosen in the upmodulation group and DEGs for metabolic pathway were chosen in the downregulation group. GO enrichment analysis of selected key circRNAs that were upmodulated and downmodulated was carried out (S3, 4). We constructed the network between circRNA and potential target genes upon ceRNA regulatory mechanism without the miRNAs (Figures 6(a) and 6(b)). The DRG samples from rats in the LPS-treated and sham subgroups were taken to verify the bioanalytical findings. qRT-PCR of the predicted two upmodulated circRNAs (Esrrg and Map3k3) and two downmodulated circRNAs (Dgkb and Atp2a2), which were selected depending on the aforementioned criteria, was performed (Figures 6(c) and 6(d), *P* < 0.01).

### 3.5. Validation of Participation in the Focal Adhesion and Metabolism Pathways

**Figure 7:**
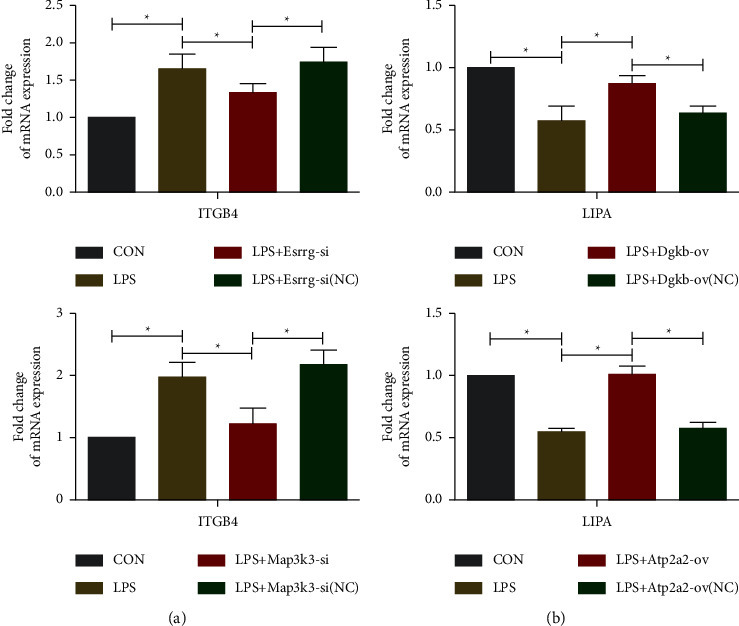
Induction of DRG by LPS mimics the in vitro NP model. Small interfering RNAs and overexpression plasmids knocked out and overexpressed circRNAs, followed by qRT-PCR validation to upregulate NP-related circRNAs (Esrrg and Map3k3) to regulate ITGB4 and downregulate NP-related circRNAs (Dgkb and Atp2a2) to regulate LIPA. (^*∗*^*P* < 0.05, *n* = 6/group, one-way ANOVA with a post hoc Tukey method).

**Figure 8:**
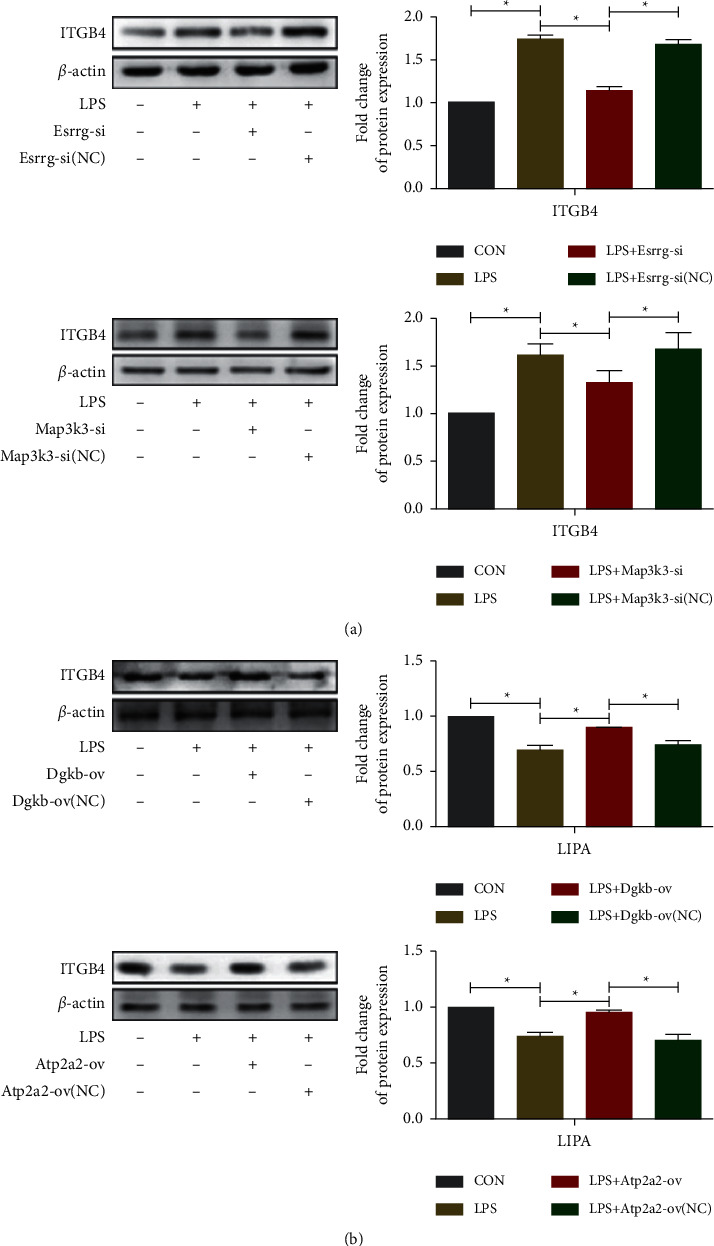
Small interfering RNAs and overexpression plasmids knocked out and overexpressed circRNAs, followed by WB validation to upregulate NP-related circRNAs (Esrrg and Map3k3) to regulate ITGB4 protein and downregulate NP-related circRNAs (Dgkb and Atp2a2) to regulate LIPA protein. (^*∗*^*P* < 0.05, *n* = 6/group, one-way ANOVA with a post hoc Tukey method).

After the basic analysis of circRNAs, two upregulated circRNA Esrrg and circRNA Map3k3 were predicted to coregulate the adhesion-related molecule Integrin Subunit Beta 4 (ITGB4) (S5). To verify the relationship between upregulated circRNAs (Esrrg and Map3k3) and ITGB4. Using LPS to establish an in vitro model of NP, it was discovered that the gene and protein expression levels of ITGB4 were substantially elevated in the LPS group (Figures 7(a) and 8(a), *P* < 0.05). Knockdown of Esrrg and Map3k3 using small interfering RNA significantly reduced LPS-induced elevation of ITGB4 gene/protein expression (*P* < 0.05). Downregulated circRNA Dgkb and circRNA Atp2a2 were predicted to coregulate the lipid metabolism-related molecule Lipase A (LIPA) (S6). In order to clarify the relationship between circRNAs (Dgkb and Atp2a2) and LIPA. The findings illustrated a decrease in the expression level of the LIPA gene and protein in the LPS group (*P* < 0.05). Overexpression of Dgkb and Atp2a2 remarkably suppressed the reduction of LIPA gene and protein expression mediated by LPS intervention (Figures 7(b) and 8(b), *P* < 0.05).

## 4. Discussion

CircRNAs are noncoding RNAs that have recently been discovered to exert functional properties. Over the last several years, researchers have discovered aberrant expression patterns of circRNAs in NP [4, 12]. In addition to being recognized as major modulators of NP, the NP-associated circRNAs are implicated in diverse biological activities. According to the information we reviewed, deregulation of specific circRNAs could cause the development of NP. A specific circRNA modulated by sponging its target miRNAs modulated the homologous downstream mRNAs and proteins in NP [13].

In this study, we try to construct the potential ceRNA network during NP by integrated time course circRNA and gene expression data at a system level. In total, 206 differentially expressed circRNAs were detected. Functional enrichment analysis of the parental genes showed these DECs were signal transduction, neuronal projections, and adhesion related. Through construct circRNA-miRNA-mRNA network and functional annotation of target genes in the ceRNA network, two pathways like focal adhesion and metabolism were considered to be regulated by DECs functioned as ceRNA. CircRNAs with elevated levels may be implicated in the modulation of NP advancement via the focal adhesion pathways. Downregulated circRNAs might modulate the progression of NP through metabolic pathways.

Whether the two pathways like focal adhesion and metabolism are truly significant altered during NP. We use two favorable bioinformatic tools to check these findings. By GSVA analysis, we found pathways about ECM interaction and immune system were with elevated activity after LPS treatment, while metabolism-related especially fatty acid metabolism was downregulated. Furthermore, we performed WGCNA analysis to construct coexpressed gene modules. Two modules were found to be with statistically altered expression through time course. Hub genes in these 2 modules were related to focal adhesion and metabolism. These two results are quite consistent with the findings in circRNA-ceRNA network analysis. So, we can infer that circRNA plays an important role in the regulation of NP.

The focal adhesion pathway performs an instrumental function in cell proliferation, migration, and patient survival, and it has been proposed as a potential treatment target. Several studies have demonstrated that focal adhesion signaling regulates the regeneration of axons in peripheral neurons [14]. Advillin is implicated in somatosensory neuronal subtype-specific axonal regeneration and NP, and it has been demonstrated to be substantially linked to newborn focal adhesion proteins [15] ITGB4, which belongs to the integrin gene family, is implicated in the migration of tumor cells and has been shown to be a good prognostic indicator in patients with colon cancer [16]. In this study, ITGB4 was a hub gene during NP by WGCNA analysis. Two upregulated circRNAs regulations of ITGB4 were found to be associated with focal adhesions, and the present ceRNA network analysis might have integral functions in focal adhesion-induced NP. Furthermore, for the treatment of NP, it is possible that ITGB4 will prove to be a new and viable therapeutic target. Nevertheless, additional experimental validation is necessary in order to corroborate this conclusion.

Research reports have illustrated that metabolic dysregulation performs a central function in NP and that there is cross-talk between metabolic, inflammatory, and immune responses in NP [17]. Previous studies have shown that NP led to metabolic changes in serum and spinal cord; changes in thalamic neurotransmitter metabolism have also been reported [18]. Functional enrichment analysis in bioinformatic investigations on NP revealed that the majority of the downmodulated genes are linked to pathways including positive modulation of protein kinase B signaling, phospholipid metabolic activities, and metabolism of xenobiotics by cytochrome P450 [19]. In this experiment, we found that LIPA was a hub gene by WGCNA analysis and significantly associated with NP-related downregulated circular RNAs, thus demonstrating that metabolic pathways affect NP. LIPA may be an effective target for the treatment of NP, which requires more in-depth mechanism studies.

In summary, this research presents a comprehensive viewpoint on the mRNAs and circRNAs expression in NP. The findings provide convincing evidence that the detected circRNAs and mRNAs are possible biological markers for NP and that the circRNAs linked to NP were engaged in adhesion and metabolic pathways. Nonetheless, additional research reports on the target validation and functional analysis of these circRNAs are required before clear evidence can be provided about the modulatory mechanisms that act on circRNAs in NP. To fully comprehend the pathogenic functions of circRNAs, the following should be done: (1) additional validation of circRNA expression, not just in cellular models; (2) in-depth study of their roles in NP through in vivo trials; (3) examining the molecular and cellular mechanisms that underly circRNAs function and evaluating their role as therapeutic targets; and (4) using these indicators in the diagnosis and prognosis of NP.

## Figures and Tables

**Table 1:** Primer sequences for a quantitative reverse transcription-polymerase chain reaction.

Gene ID	Primer sequence	
	Forward (5′-3′)	Reverse (5′-3′)
chr13:106507145-106507561+	TTCAAGAGGACAATTCAAGGCTTC	TTCCGTCTTGATGAAGGACGA
chr10:94292692-94314584+	ATCCCAAGACAGAAACCATATGAAC	TCTTCATGGTCTCATATCCAGACAC
chr6:57758533-57852070+	TTTCCATTGGCGTGAATGTG	TGTTCAGGCAGAGATTGCAGTAG
chr12:39564848-39567224+	GTGGAAATTGCTGGTTTTGGC	GCATTGGCTACCAATATAAGCAGA
GAPDH	ACAGCAACAGGGTGGTGGAC	TTTGAGGGTACAGCGAACTT

## Data Availability

All data, models, and code generated or used during the study are included within the article.
